# A complex intervention to promote prevention of delirium in older adults by targeting caregiver’s participation during and after hospital discharge – study protocol of the TRAnsport and DElirium in older people (TRADE) project

**DOI:** 10.1186/s12877-021-02585-0

**Published:** 2021-11-16

**Authors:** Christoph Leinert, Simone Brefka, Ulrike Braisch, Natascha Denninger, Martin Mueller, Petra Benzinger, Juergen Bauer, Anke Bahrmann, Norbert Frey, Hugo A. Katus, Tobias Geisler, Gerhard Eschweiler, Jochen Klaus, Thomas Seufferlein, Konrad Schuetze, Florian Gebhard, Jens Dreyhaupt, Rainer Muche, Kathrin Pahmeier, Janine Biermann-Stallwitz, Juergen Wasem, Lena Flagmeier, Dhayana Dallmeier, Michael Denkinger

**Affiliations:** 1 Agaplesion Bethesda Clinic Ulm, Zollernring 26, 89073 Ulm, Germany; 2 Geriatric Center Ulm/ Alb-Donau, Ulm, Germany; 3 Institute for Geriatric Research, Ulm University, Ulm, Germany; 4 Institute of Epidemiology and Medical Biometry, Ulm University, Ulm, Germany; 5 Center for Research, Development and Transfer, Rosenheim Technical University of Applied Sciences, Rosenheim, Germany; 6 International Graduate Academy, Institute for Health and Nursing Science, Medical Faculty, Martin-Luther-University Halle-Wittenberg, Halle (Saale), Germany; 7 Faculty of Applied Health and Social Sciences, Rosenheim Technical University of Applied Sciences, Rosenheim, Germany; 8 Center for Geriatric Medicine, Agaplesion Bethanien Krankenhaus Heidelberg, Heidelberg University, Heidelberg, Germany; 9 Institute of Health and Generations, Faculty of Social and Health Studies, University of Applied Sciences Kempten, Kempten, Germany; 10 Department of Cardiology, Angiology and Pneumology, University Hospital of Heidelberg, Heidelberg, Germany; 11 Department of Internal Medicine, University Hospital of Heidelberg, Heidelberg, Germany; 12 Department of Cardiology, University Hospital Tuebingen, Tuebingen, Germany; 13 Geriatric Center at the University Hospital Tuebingen, Tuebingen, Germany; 14 Department of Medicine I, University Hospital Ulm, Ulm, Germany; 15 Department of Trauma-, Hand-, and Reconstructive Surgery, Ulm University, Albert-Einstein-Allee 23, 89081 Ulm, Germany; 16 Institute for Health Care Management and Research, University of Duisburg-Essen, Essen, Germany; 17 AOK – Allgemeine Ortskrankenkasse Baden-Wuerttemberg, Stuttgart, Germany; 18 Department of Epidemiology, Boston University School of Public Health, Boston, USA

**Keywords:** Caregiver, Complex intervention, Delirium, Dementia, Discharge, Family, Geriatrics, Older adults, Transfer, Transport

## Abstract

**Background:**

Among potentially modifiable risk factors for delirium, transfers between wards, hospitals and other facilities have been mentioned with low evidence. TRADE (TRAnsport and DElirium in older people) was set up to investigate i) the impact of transfer and/or discharge on the onset of delirium in older adults and ii) feasibility and acceptance of a developed complex intervention targeting caregiver’s participation during and after hospital discharge or transfer on cognition and the onset of delirium in older adults.

**Methods:**

The study is designed according to the guidelines of the UK Medical Research Council (MRC) for development and evaluation of complex interventions and comprises two steps: development and feasibility/piloting. The development phase includes i) a multicenter observational prospective cohort study to assess delirium incidence and cognitive decline associated with transfer and discharge, ii) a systematic review of the literature, iii) stakeholder focus group interviews and iv) an expert workshop followed by a Delphi survey. Based on this information, a complex intervention to better and systematically involve family caregivers in discharge and transport was developed. The intervention will be tested in a pilot study using a stepped wedge design with a detailed process and health economic evaluation. The study is conducted at four acute care hospitals in southwest Germany. Primary endpoints are the delirium incidence and cognitive function. Secondary endpoints include prevalence of caregiver companionship, functional decline, cost and cost effectiveness, quality of discharge management and quality of admission management in admitting hospitals or nursing homes. Data will be collected prior to discharge as well as after 3, 7 and 90 days.

**Discussion:**

TRADE will help to evaluate transfer and discharge as a possible risk factor for delirium. In addition, TRADE evaluates the impact and modifiability of caregiver’s participation during patient’s transfer or discharge on delirium incidence and cognitive decline providing the foundation for a confirmatory implementation study.

**Trial registration:**

DRKS (Deutsches Register für klinische Studien) DRKS00017828. Registered on 17th September 2019. Retrospectively registered.

**Supplementary Information:**

The online version contains supplementary material available at 10.1186/s12877-021-02585-0.

## Background

Delirium is a growing medical burden especially for hospitalized older adults with incidences of up to one third among those 70 years of age or older [1]. In a meta-analysis with 42 studies delirium in hospital and post-acute care was associated with increased post discharge mortality and institutionalization rates [2]. Risk factors for delirium are multidimensional and include non-modifiable aspects such as age, acute illness or pre-existing dementia [3]. Others, like major surgery, anesthesia, immobilization during inpatient treatment, drug side effects and several psychological, social and environmental factors are linked to acute hospital care itself and potentially modifiable [4–7]. Delirium is associated with complications such as falls, nosocomial infections and subsequent loss of autonomy. Delirium prevention and treatment is, therefore, an indicator of patient safety and quality in modern health care settings [8, 9].

Most deliria occur during the first days after admission to a hospital. In addition to the acute illness, hospital and room transfers could account as triggers [10]. In retrospective cohort studies room transfers in acute care hospitals have been shown to increase delirium risk in surgical and nonsurgical patients [11, 12]. However, the impact of transportation or transfer within a hospital and/or after discharge has not been evaluated prospectively.

Considering therapeutic and preventive strategies, non-pharmacologic interventions with a focus on reorientation, minimal change in hospital staff and involvement of family caregivers are effective ways to reduce delirium incidence during hospital stay [13]. Potentially modifiable environmental factors such as providing reading glasses and hearing aids or family support can reduce the severity of delirium symptoms in nursing home residents [14]. The most prominent multicomponent non-pharmacologic intervention is the Hospital Elder Life Program (HELP), which reduced in a hospital setting cognitive decline from 26 to 8% and functional decline from 33 to 14% in a cohort of older persons [15]. A meta-analysis of 14 interventional studies showed that a multicomponent intervention including changes in environmental factors was associated with a significant risk reduction for incident delirium of 0.53 (95% confidence interval (CI) 0.38 to 0.58) [16].

The TRAnsport and DElirium in older people (TRADE) study aims to evaluate the impact of transfers including the discharge process on subsequent delirium incidence and cognitive decline. Furthermore, a complex intervention will be developed that involves caregivers during and after transitional care to prevent delirium using non-pharmacologic strategies. This intervention will be tested in a pilot study including a process and health economic evaluation.

## Methods

### Study design

**Fig. 1:**
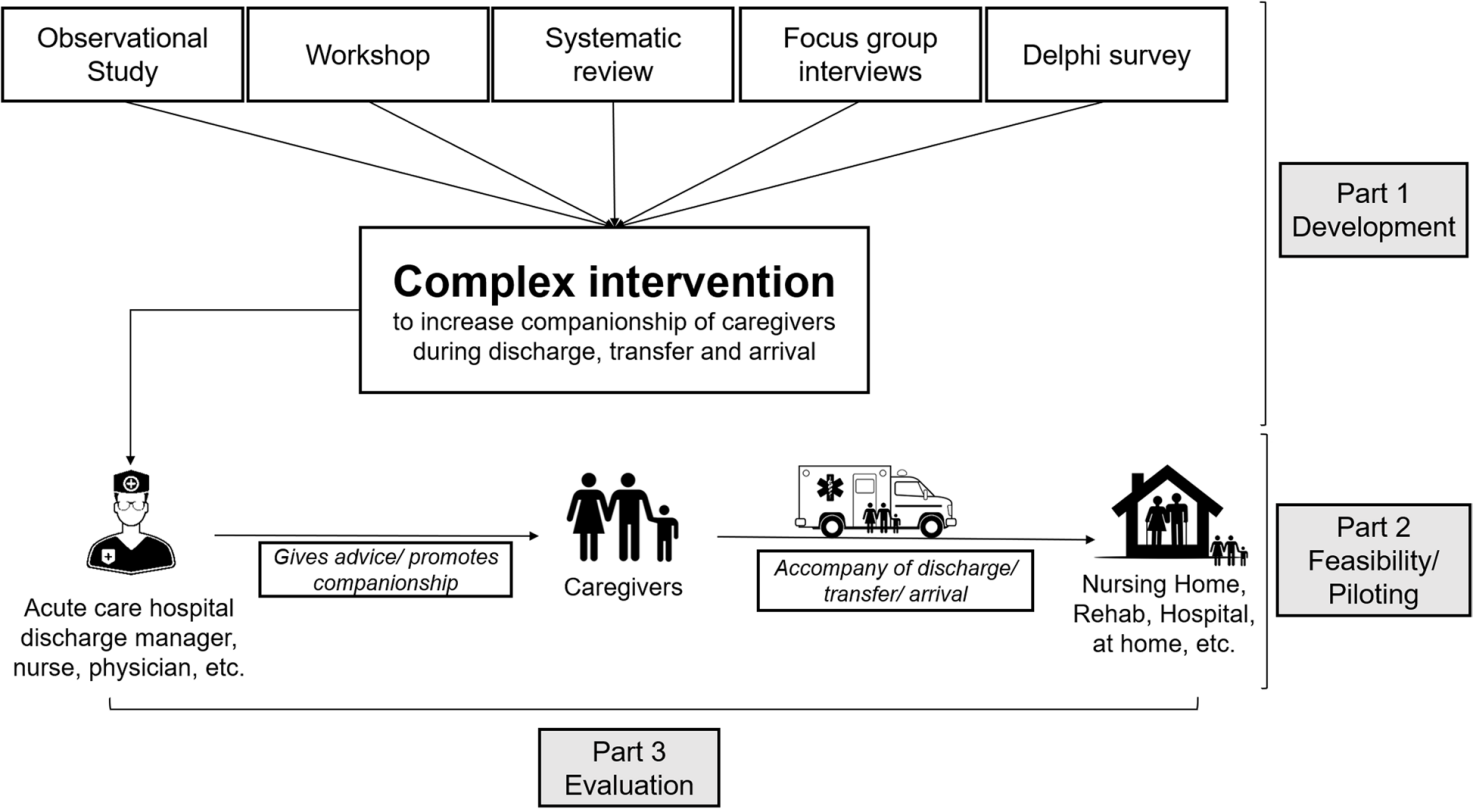
Project overview. TRADE consists of three parts

The TRADE project comprises the first two steps of the Medical Research Council (MRC) framework for the development and evaluation of complex interventions: development and feasibility/piloting [17, 18]. TRADE consists of three study parts shown in Fig. 1:

### Development (part 1)

The development phase of TRADE includes identification of existing evidence, identifying and/or developing of intervention theory, modeling processes and the definition of outcome measures. For this purpose, a prospective observational study, a systematic review, focus groups, an expert workshop and a Delphi survey have been performed.

### Observational cohort study

A prospective observational cohort study was conducted to identify the impact of transfer to another hospital department or discharge from the hospital on the incidence of delirium and cognitive decline. Participants were recruited at departments for internal medicines at four study centers at three university hospitals: Heidelberg: Department of Cardiology, Tuebingen: Departments of Cardiology and Gastroenterology, Ulm: Departments of Gastroenterology and Trauma Surgery and one academic geriatric hospital (Agaplesion Bethanien Krankenhaus Heidelberg, associated to Heidelberg University) located in southwest Germany. The observational study was one of the main components for the development of the pilot intervention. Study modalities (e.g. inclusion/exclusion criteria, assessments) were comparable to the piloting intervention, described in detail below. The observational study was performed from August 2019 till February 2020 and included 245 participants.

### Systematic review

The aim of the systematic search and review was to identify the existing evidence for risk factors for delirium as well as for effective non-pharmacological intervention components to prevent delirium associated with a change of location or transfer for patients aged 65 years or older.

### Focus group interviews

Focus group interviews were conducted with involved health care professionals of the participating hospitals and with transport providers to explore the local discharge and transitional care procedures, existing practice, problems and needs. Furthermore, people involved in the discharge process should be identified and targeted for intervention.

### Expert workshop

The results of the three previous steps (observation study, review, focus group interviews) were synthesized in a structured expert workshop using nominal consensus techniques involving health care professionals to identify and develop a first draft of intervention components and implementation strategies.

### Delphi survey

The draft from the expert panel was consented in an online-Delphi survey involving health care professionals participating in the workshop, external experts of delirium and caregiver representatives. In this modified Delphi process, experts were asked to make comments or further suggestions [19]. Since there was already a strong agreement in the first round with only a few minor comments, we refrained from implementing a second round.

### Main results of the development part

Based on this development process a complex intervention has been designed to achieve the a priori target of promoting caregiver companionship of participants during and after transitional care.

A logic model was used to visualize the whole collected information and theoretical foundations of the development phase. This was used to represent what is going to be done, how the individual components are interrelated and what results are expected [20].

Detailed results of the whole development of the complex intervention will be published elsewhere.

**Fig. 2:**
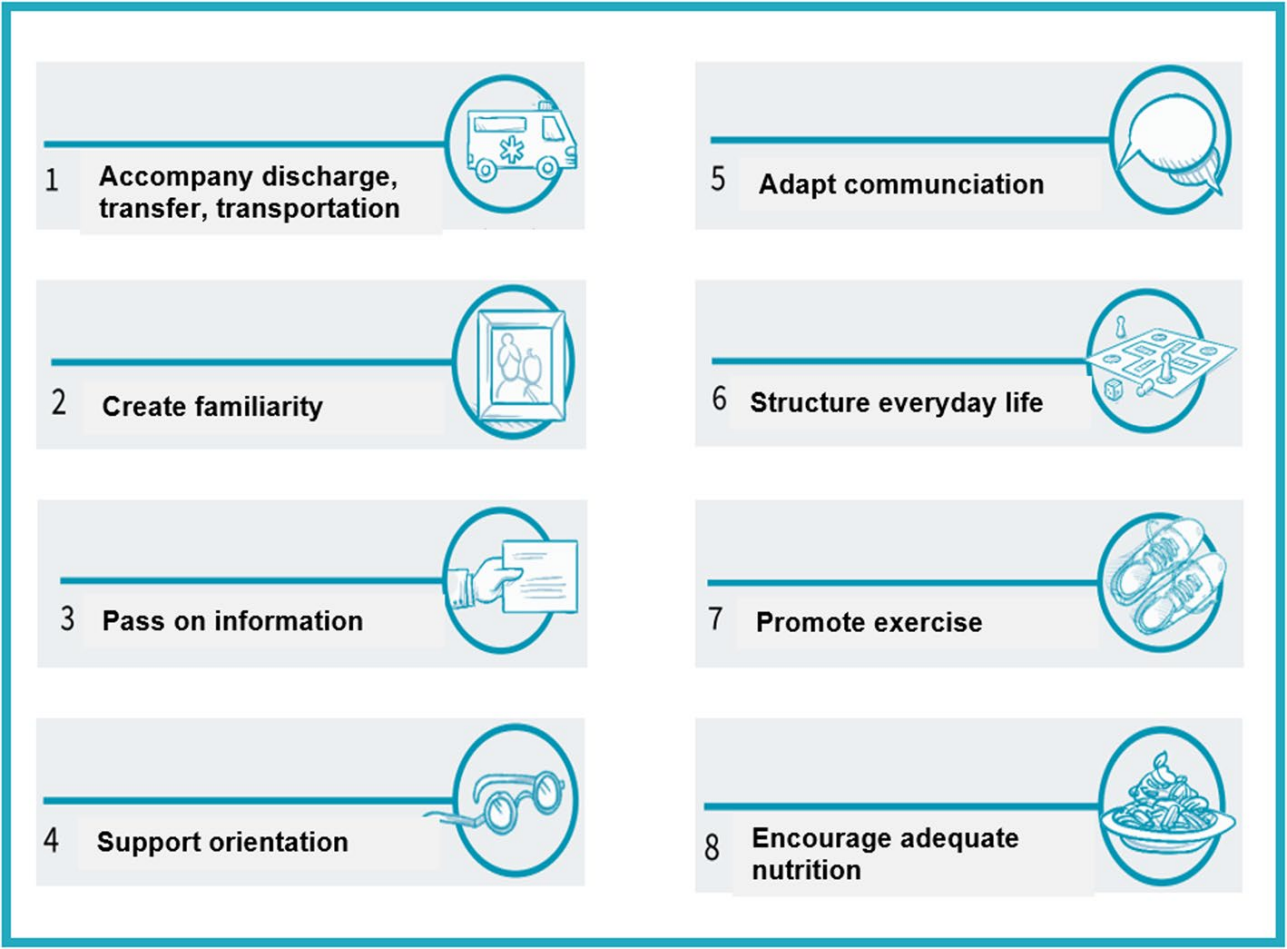
Delirium prevention and intervention strategies with eight major items (8-item-program)

Tailormade information materials for relatives or confidents, discharge managers, social service professionals and health care professionals were developed. The results are summarized as follows: The major aim of the intervention is to empower caregivers to support the persons they care for during and after transfer and discharge from the hospital, including in some cases the arrival at a new institution. In addition to maximizing the time of companionship other seven important elements were identified building the 8-item program that is shown in Fig. 2 as part of our intervention booklet: accompany discharge, transfer and transportation, create familiarity, pass on information, support orientation, adapt communication, structure everyday life, promote exercise and encourage adequate nutrition.

Analysis of focus groups and expert workshops confirmed the a priori hypothesis that the implementation of the intervention should not only focus on hospital staff, but also on patients and caregivers alike. Before starting the intervention, gatekeepers will be identified in all participating centers in order to record the local structures and discharge processes at the beginning and at the end of the intervention phase, to be able to draw a comparison. With beginning of the intervention, all identified healthcare personal involved in the discharge process will be trained. Because discharge structures differ from hospital to hospital, multipliers, called champions, will be identified and trained at a maximum of 3 weeks before start of the intervention in each participating center to advise the rest of the staff involved in discharge procedures such as physicians, ward nurses, social workers, ward secretaries and others. This *train the trainer* approach consists of several steps. In a first step awareness for the study, the relevance of delirium and the motivation to participate in the intervention has to be promoted. In a second step, champions will be constantly supported to adhere to the intervention by regular video or telephone meetings with the project team. The other health care professionals will be also informed about delirium and the interventions in a short training session.

In addition to the focused training, discharge managers and associated clinical disciplines will be provided with educational material as well as with prepared communication strategies to facilitate focused briefing to caregivers and patients. The educational materials consist of the following:

- A handbook for discharge managers and health care professionals: It summarizes general information on delirium risk factors and clinical impact in acute care hospitals. In addition, it outlines the 8-item-program and supports professionals providing a conversation guide for structured information provision to patients and primary caregivers.
- A check list for the discharge procedure for health care professionals.
- An educational flyer for primary caregivers: It includes all essential information on non-pharmacologic intervention strategies in the 8-item-program. Primary caregivers will be encouraged to deliver the intervention and support patients around hospital discharge. Flyers will be also used to generate awareness in other professionals e.g. transport service staff.
- A “Dos and Dont’s” poster for health care professionals: They will be placed in highly frequented places in the participating departments and present important principles of delirium friendly discharge and links to further information materials supplied by the TRADE study.
- “1-min-information” posters for health care professionals (following the “One minute Wonder” by Schmidt & Krueger [21, 22]): These are different posters that are displayed at highly frequented places in the participating departments and where persons wait for a maximum of 1 min (e.g. coffee machine). They contain brief information on delirium in general and delirium friendly discharge strategies with focus on one topic on every poster. Posters will be exchanged weekly to ensure ongoing attention to the project and to refresh knowledge on the topics.
- Online information videos for health care professionals as well as for caregivers with expert interview concerning delirium in general and in the context of discharge and transfer.

**Fig. 3:**
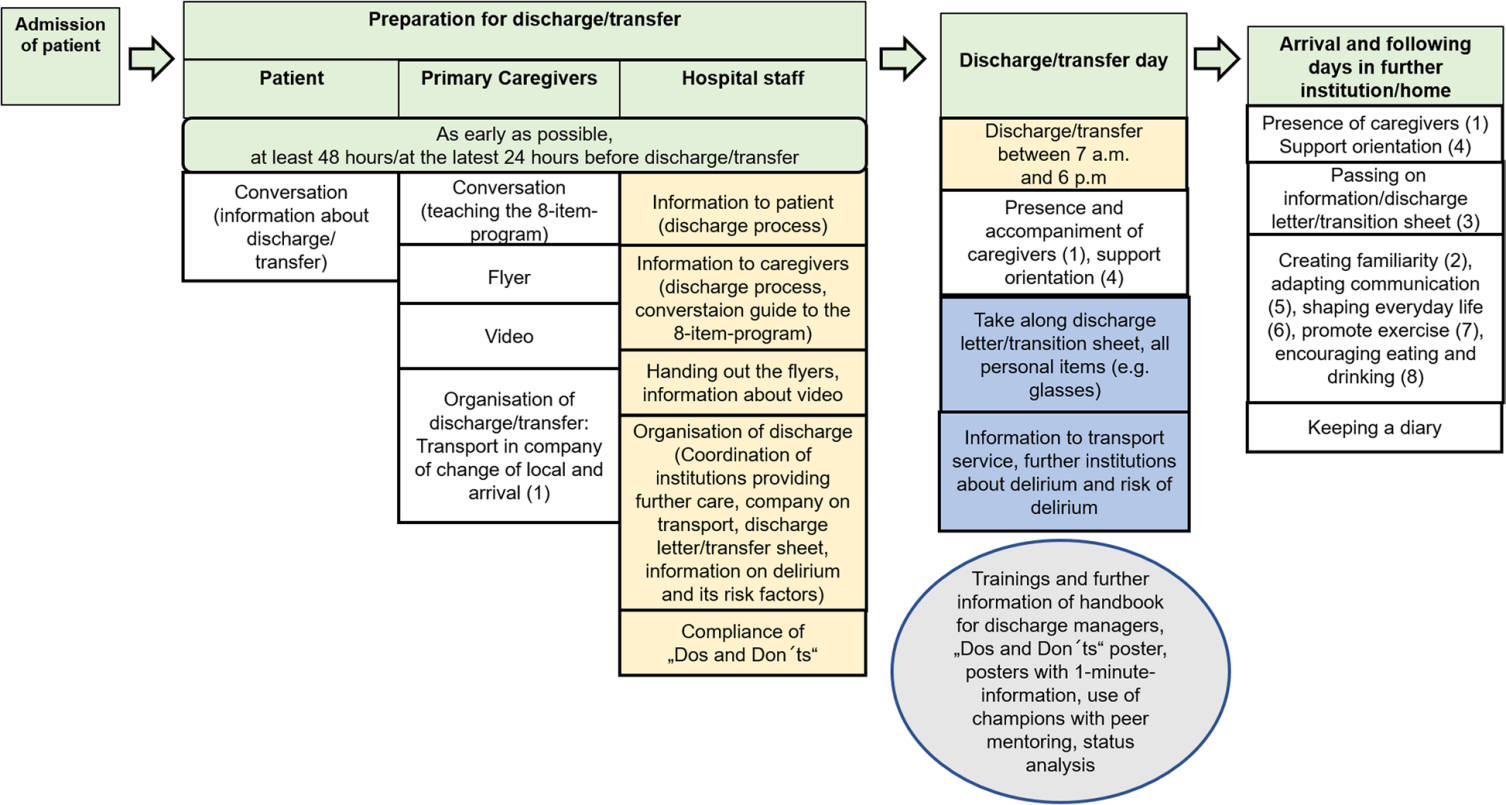
Elements of intervention in the timeline of discharge management. Legend: Green boxes: timeline of discharge planning; white boxes: involvement of caregivers, number in brackets: aspect of 8-item-program; blue boxes: involvement of nurses/ ward secretaries; orange boxes: involvement of health care professional depending on the procedures of hospital (responsibility of nurses/physician/discharge manager/social services/etc.); grey circle: Intervention elements

A summary of key components of the intervention in the context of the participant’s hospital treatment timeline can be found in Fig. 3:

### Feasibility and piloting (part 2)

In the second part, a pilot study is conducted in the four participating centers using a stepped-wedge design in order to test the feasibility of the complex intervention for increasing caregiver companionship of participants during transitional care and its impact on delirium prevention.

Due to severe acquired respiratory syndrome-corona virus 2 (SARS-CoV2)-pandemic the number of encounters with the participants had to be reduced compared to the observational cohort study and the start of the intervention had to be postponed from October 1st 2020 to April 1st 2021. Baseline and follow-up assessments were adapted based on the experiences of the development phase. In total, 500 participants are planned to be recruited, equally distributed across the four intervention sites. This includes the observed dropout rate of approximately 20%.

### Study population

All patients 70 years or older admitted to the participating wards will be informed about the TRADE project. In every study center, the TRADE Study team consists of trained study nurses and research assistants, supported by study physicians. Once consent is obtained, participants will be assessed by trained assessors.

Admissions to acute care will be regularly screened for eligibility for the study. In addition, awareness of patients and caregivers will be raised by putting up advertising posters and adding an informative flyer about the TRADE Study to the regular hospital admission folder which also refers to more information on the website.

In addition to patients, their primary caregivers will also be included in the study. We defined primary caregivers as follows: everybody with a close personal relationship to the patient such as partners and other family members but also close friends, confidents, neighbors or long-term legal guardians as identified by the patients. During the observational part of the study primary caregivers were asked for participation whenever possible. As caregiver’s participation is a key element for a successful intervention, their participation is mandatory during the pilot intervention part of the study.

### Inclusion criteria

All inpatients of participating departments aged 70 years or older that are planned for discharge within the next 4 days were eligible for the observational study. Considering the rapid changes observed in the clinical course during hospitalization the allowed time from inclusion in the study till discharge was reduced in the pilot study from four to three days.

### Exclusion criteria

Patients with insufficient knowledge of the German language are excluded. For patients who are unable to provide consent because of cognitive deficits an informed consent will be acquired from their legal representatives. If no legal representative is available, patients have to be excluded from the study. Patients with a need for palliative care medicine and an estimated prognosis of less than 3 months of survival are also excluded from the study.

### Patients with cognitive impairment

According to representative cohort studies on inpatients in German hospitals approximately 40% of examined patients will have slight or severe cognitive impairment [23]. Patients with cognitive impairment have a higher risk to develop delirium, which at the same time is known to promote persistent progression of cognitive impairment [24]. Therefore, patients with cognitive impairment especially benefit from delirium prevention strategies.

Supplementary Figure 1 provides standardized algorithm for inclusion/exclusion to address the challenge to include this specific group.

### Patients’ withdrawal

Patients are allowed to withdraw their participation in the study at any given point of time. Reasons for discontinuation will be assessed if allowed by the patient.

### Intervention

As described above the intervention has been created in a development process with multiple components. The involvement of primary caregivers is a key element to develop a multimodal non-pharmacological delirium intervention in a cost-efficient and easy implementable way.

### Outcomes and measurements

#### Primary outcomes

Primary outcomes are delirium incidence and cognitive function [25].

#### Delirium

When screening for delirium, anamnestic information on acute onset and fluctuating course is essential for delirium detection, but can be difficult to acquire in a single contact with the participant. Therefore, delirium screening strategy in TRADE combines information from different sources: patient, caregiver, and nurse. Delirium is screened by interviewing the patient, the primary caregiver and the nursing staff at baseline and on day 3, 7 and 90 after discharge. In the intervention part of the study delirium screening on day 3 after discharge will only be performed in a telephone interview with the primary caregiver.

As main delirium screening tool for assessing the patient the German version of the Confusion Assessment Method (CAM) is being used [26, 27]. This gold standard of delirium detection has been adapted over time to several sub-assessments preserving the four key elements in detecting inattention, disorganized thinking, alteration of consciousness and acute onset/ fluctuating course. In the observational cohort study we use the I-CAM-S Version combining a double check with ICD-10 criteria (I-CAM) and a delirium severity score (CAM-S) as described by Sánchez et al. [7, 28, 29]. In the interventional part of the study the 3-min diagnostic interview CAM version (3D-CAM) has been added [30]. 3D-CAM facilitates assessment for the raters by detailed instructions and closed questions. For the caregivers the Family Confusion Assessment Method (FAM-CAM) will be used [31]. The five-item Nursing delirium screening scale (Nu-DESC) allows to rate attending nurses’ observations concerning disorientation, inappropriate behavior and communication, hallucinations and psychomotor retardation over a 24-h period [32]. For the interventional part of the study two easy to use screening instruments for delirium were added to the assessment battery: The bedside confusion scale (BCS) as a simple and short test for delirium that can be derived from 3D-CAM assessment and focuses on the items psychomotor disturbance and awareness [33]. In addition, attending nurses and primary caregivers will be asked the *Single Question in Delirium* assessment (SQiD): ‘Do you think [name of patient] has been more confused lately?’ [34].

The combined endpoint is defined as follows: delirium is present if signs of delirium are reported in one of the delirium assessments regardless of the source (patient, caregiver, nurse). Subgroup analysis evaluating the role of patient assessment versus caregiver/nurse assessments are considered if feasible.

#### Cognitive function

For assessment of cognitive function three different versions of the Montreal Cognitive Assessment (MoCA) Version 7 is used on baseline and on day 7 and 90 of the follow-up, respectively [35]. The MoCA is a brief cognitive screening test well established in assessment of older persons. Its advantage against other short cognitive tests is the assessment of multiple cognitive domains including visuo-spatial ability, attention, executive functions, memory, language, abstraction and orientation [35].

#### Secondary outcomes

Secondary outcomes are mainly proxies for physical function such as activities of daily living (ADL) and history of falls, antipsychotic medication use, mortality and new admissions to residential care.

For basic ADLs the Barthel Index and for instrumental ADLs the Lawton Index are used [36–38]. Mobility is assessed via the Rivermead mobility index [39]. It was chosen because it can capture the wide mobility range that is expected in geriatric medical inpatients without relevant ceiling or floor effects.

A complete medication list including medication type, dose and frequency, is recorded at baseline and at every following visit. This opens the opportunity to track the change in antipsychotics as well as other medication classes. As anticholinergic burden is a relevant risk factor of delirium incidence in older adults, medications will be assessed under this aspect [40, 41].

Companionship of caregivers during and 7 days after discharge will be measured by documenting the number of visits and amount of time spend by the caregivers with their confidents. Additional rate of application of the different protocols of the 8-item-program will be recorded.

For health economic evaluation, data of all participants who are insured by a specific health care insurance (Allgemeine Ortskrankenkasse Baden-Wuerttemberg [AOK]) and have consented to the transfer of their routine data will be analyzed separately. Participants insured by AOK are estimated to form about 1/3 of the study population. Routine data will be analyzed 1 year before and 3 months after transfer/discharge. Costs and cost-effectiveness (outcome delirium prevented) will be evaluated from a social health insurance perspective. In addition, the intervention-specific resource use of patients, relatives and hospital staff will be determined.

#### Covariates

Basic sociodemographic patient information includes age, gender, weight, height, vital signs, marital status, family network and regular social contacts, living arrangement, immigration background, education level, occupation, advanced directive and power of attorney status, nicotine consumption, alcohol consumption, falls, use of walking aids, and care level.

Sensory function is assessed using the visual acuity and whisper test as well as through self-report [42, 43]. Handgrip strength is measured with the Jamar® Hydraulic Hand Dynamometer [44]. Subjective memory impairment and history of previous episodes of delirium are also addressed [45]. Comorbidities and personal medical history are obtained by asking the patient “Have you ever been diagnosed any of the following diagnosis?:”, following the read out of an adapted version of the Charlson Comorbidity Index (CCI) listing [46]. In addition, all diagnoses on the discharge summary are extracted. Depression and anxiety are measured by the Patient Health Questionnaire (PHQ-4) [47]. Sleep quality is quantified based on the Pittsburgh Sleep Quality Index (PSQI) [48]. Frailty is assessed with the Clinical Frailty Scale of the Canadian Study of Health and Aging (CSHA Clinical Frailty Scale) [49]. Pain is measured using the Numeric Rating Scale (NRS) [50].

Primary caregivers are interviewed concerning pre-existing cognitive deficits using the Informant Questionnaire on Cognitive Decline in the Elderly (IQCODE) [51]. Because of the SARS-CoV2-pandemic a questionnaire concerning psychological and social burden for patients and primary caregivers was set up and integrated at T2 in the intervention study.

Following laboratory measurements are collected at baseline from the documentation: leukocytes, mean cellular volume, hemoglobin, thrombocytes, sodium, potassium, creatinine, urea, alkaline phosphatase, liver function test, HbA1c, total amount of protein/albumin, NT-proBNP, TSH, glucose, C-reactive protein. No study-specific blood samples are taken.

Concerning process evaluation, specific questions are included into baseline and follow-up assessments that will characterize modalities and quality of discharge and transportation planning and conduct.

Table 1 gives a summary of the enrollment, intervention and assessments planned for the TRADE study.

**Table 1:**
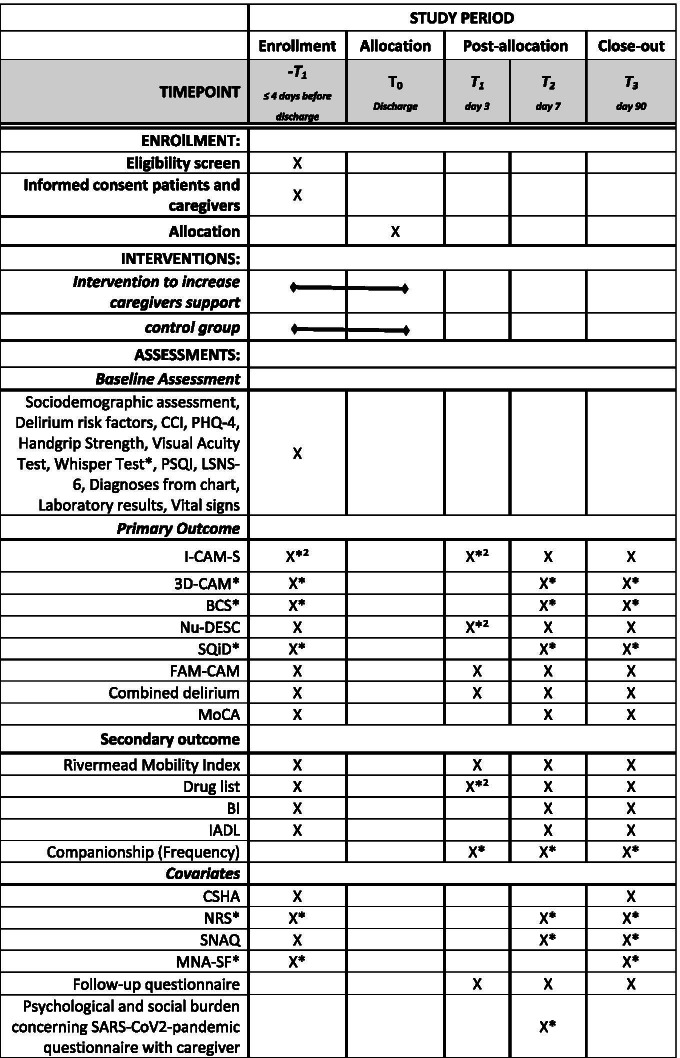
Schedule of enrollment, interventions and assessments for TRADE study

Legend: *-marked items will only be conducted in interventional part, *^2^-marked items will only be conducted in observational part. Charlson Comorbidity Index (CCI), Patient Health Questionnaire 4 (PHQ-4), Numeric Rating Pain Scale (NRS)*, Pittsburgh Sleep Quality Index (PSQI), Simplified nutritional appetite questionnaire (SNAQ), Mini Nutritional Assessment-Short Form (MNA-SF)*, Lubben-6 Social Network Skala (LSNS-6), Clinical Frailty Scale (CSHA), Informant Questionnaire on Cognitive Decline (IQCODE), Delirium risk factors (including alcohol, history of falls, sensory impairment, subjective cognitive decline, pain, history of delirium), ICD-10 supplemented Confusion Assessment Method Severity Score (I-CAM-S), 3-Minute Diagnostic Interview for Confusion Assessment Method defined Delirium (3D-CAM) *, Bedside Confusion Scale (BCS)*, Montreal Cognitive Assessment (MoCA), Nurses Delirium Rating Scale (Nu-DESC), Single Question in Delirium (SQiD) with Nurse and Caregiver*, Family-Confusion Assessment Method (FAM-CAM), Barthel Index (BI), Instrumental Activities of Daily Living (IADL)

### Participant ‘s timeline

From the participant’s perspective the study starts in the hospital with a detailed baseline assessment four/three days or less prior to discharge (T0). After discharge follow-up assessments are on day 3 (+/− 1 day; T1), on day 7 (+/− 1 day; T2) and day 90 (+/− 7 days, T3). While in the observational study all visits were done personally, for the pilot intervention a telephone assessment with the primary caregiver replaces the personal visit at T1 in order to reduce patient contacts because of the SARS-CoV2-pandemic. For T2 and T3, participants are still visited in the respective discharge environment. If facilities do not allow personal visits, a full telephone-based follow-up visit (T1-T3) will be performed.

Primary caregivers are interviewed at each follow-up timepoint (T1, T2, T3) by phone. Attending nurses are interviewed at each follow-up and baseline assessment personally or by phone (T0, T2, T3).

**Fig. 4:**
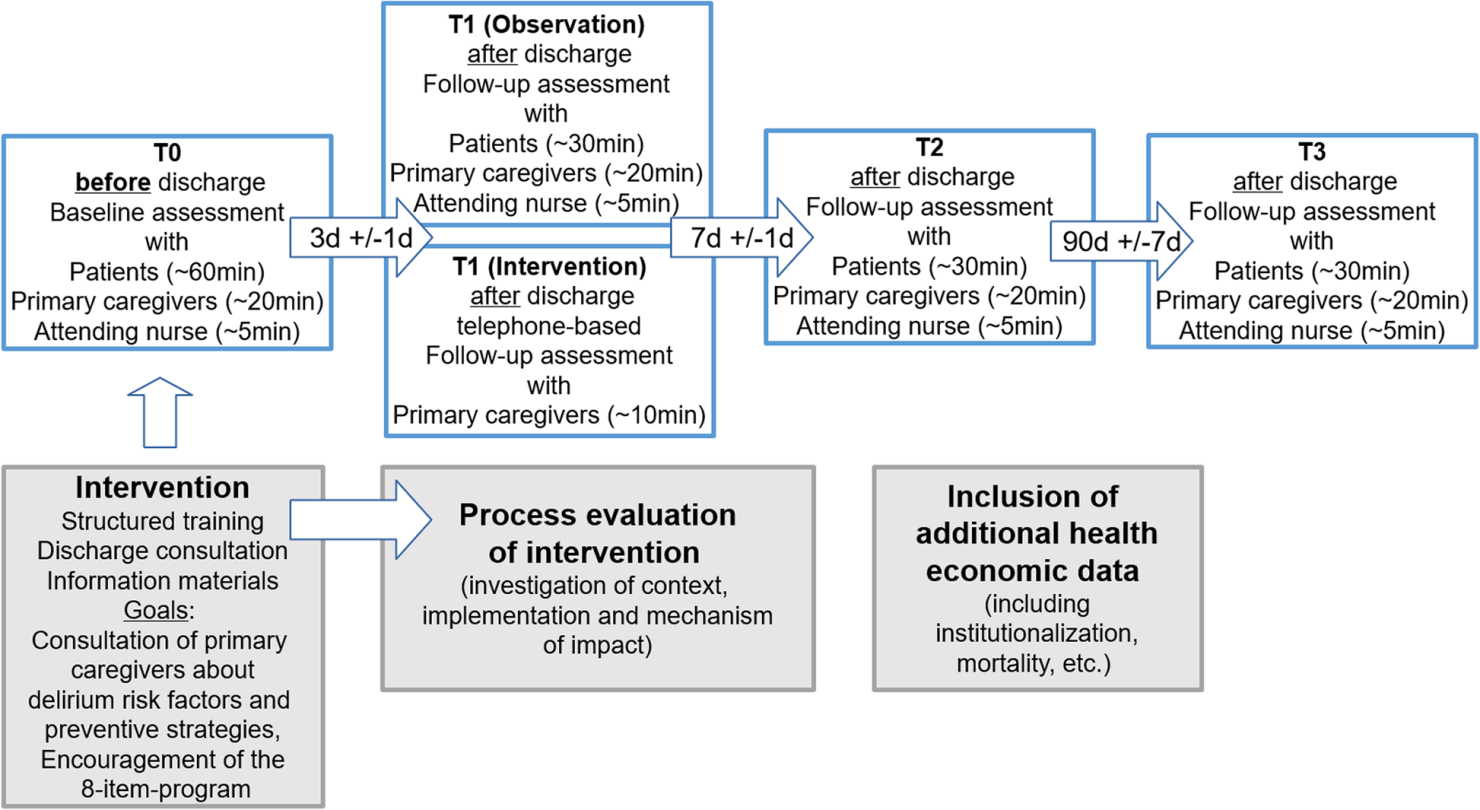
Study timelines. Legend: White boxes: Assessment time point in observation and intervention trial T0, T1, T2 and T3 with approximated assessment time per assessed group (patient/primary caregiver/nurse); grey boxes: intervention, process evaluation and additional data collection

The study timeline is summarized in Fig. 4.

### Sample size

The sample sizes for both the observational study and the pilot intervention trial were chosen based on feasibility considerations. Robust sample size estimations were not possible because of the pilot character of the study and missing preliminary information. Power analysis for the intervention study was additionally based on descriptive results from the observational part.

For the pilot study we will include 500 patients in all four study centers (125 patients each within 12 months), including an estimated dropout rate of 20%. With preliminary results of the observational study and the expected sample size of 400 (excl. drop-outs) we conducted power analyses for the primary outcomes delirium incidence and cognitive function. In the control group of the pilot intervention study we would expect an incidence of delirium of about 6% and of cognitive impairment of about 41%. Using a two-sided chi-square test at a significance level of 5% with a power of at least 80%, one should be able to show a reduction in delirium incidence in the intervention group to 0.9% and in cognitive impairment incidence to 27.7%.

Patients with an unfamiliar discharge environment and cognitive impaired patients will be preferably included, as they are the main focus of the project having a high risk of delirium.

### Stepped wedge design

For the pilot intervention study a stepped wedge design is used [52]. All clusters start without intervention. While the first cluster starts after 12 weeks, every other cluster follows after another 8 weeks until all four clinical partners have started. Please see also Table 2.

**Table 2:**
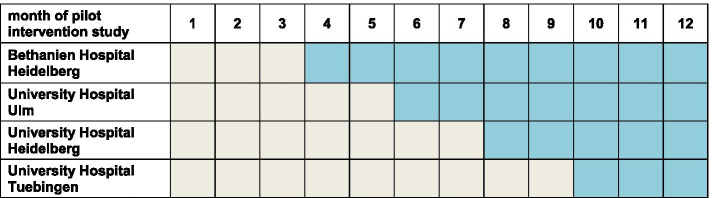
Stepped wedge design and intervention entry in the pilot intervention trial. Legend: Control phase in grey parts of the table, intervention phase in blue parts of the table

During the observational study, imbalances in the distribution of participants insured by the AOK health care insurance among the different study centers were recognized. The balanced allocation of AOK health care insurance holders is an important feature for health economic evaluation. To minimize these imbalances for the pilot intervention study, a pre-defined cluster entry as given in Table 2 was chosen rather than a randomized approach.

### Data management and monitoring

Data at baseline and follow-up visits are collected via an electronic Case Report Form (eCRF) on the web-based electronic data capture (EDC) system secuTrial® from interActive Systems GmbH. If primary entry of data into eCRF is not possible, a paper version of CRF is provided and data is transferred as soon as possible. The access to the platform is protected by an authentication process, and the transmission of data between the study centers and the secuTrial® server is protected by a secure connection using Secure Sockets Layer (SSL) encryption. All assessors and study nurses are trained in assessments and eCRF usage before participating in data collection. The members of the study central are in regular contact with the partners via weekly telephone or video conferencing and quarterly monitoring visits. Trained scientific data managers constantly double-check collected data in the eCRF database and query inconsistencies directly with the study nurses of each center. The data for primary outcome measures are additionally being checked by trained physicians. Because this ongoing monitoring of data quality a data monitoring committee is not installed.

All data from assessments and tests, as well as the clinical data and the data for the health economic analysis, are stored in a pseudonymized fashion. After the end of the study, all paper and electronic documents will be filed for at least 10 years.

### Statistical analysis

The data of the observational study will be analyzed by descriptive statistical methods and regression analyses for demographic aspects as well as for the main outcomes.

Regarding the pilot intervention study descriptive analyses and mixed models (with logit-link for binary outcome and identity-link for continuous outcome) according to Thompson et al. and Goldfeld will be conducted to account for the stepped wedge design [53, 54]. To account for missing values, we will use multiple imputation methods or sensitivity analyses [55].

Analyses will be done in total as well as stratified by study centers. Subgroup analyses for known and unknown discharge environments as well as for cognitive fit and impaired patients are planned. Statistical tests will be two sided with a 5% level of significance. Because of the explorative character of the study no adjustment for multiple testing will be done. All results of statistical tests will be interpreted as hypothesis generating and not as a statistical proof. The software Statistical Analysis Software (SAS) and R will be used for analyses.

The health economic evaluation includes patients from both the observational and interventional part. Cost and cost-effectiveness analyses will be carried out in a difference-in-differences approach including subgroup analyses (e.g. based on age, gender or cognitive status). Sensitivity analyses will be also being performed.

### Process evaluation (part 3)

The process evaluation is designed according the recommendations of the MRC guidelines of process evaluation for complex interventions [56]. The aim is to understand the context in which the intervention components are implemented, the implementation (fidelity, dose, adoptions, reach) and the mechanism of impact (delivery and response) of the intervention components and to clarify whether the intervention components were effective and if not, why not [57]. To investigate the integration, embedding and implementation of the intervention components in routine practice, the Normalization Process Theory (NPT) is used as theoretical approach [58, 59].

**Fig. 5:**
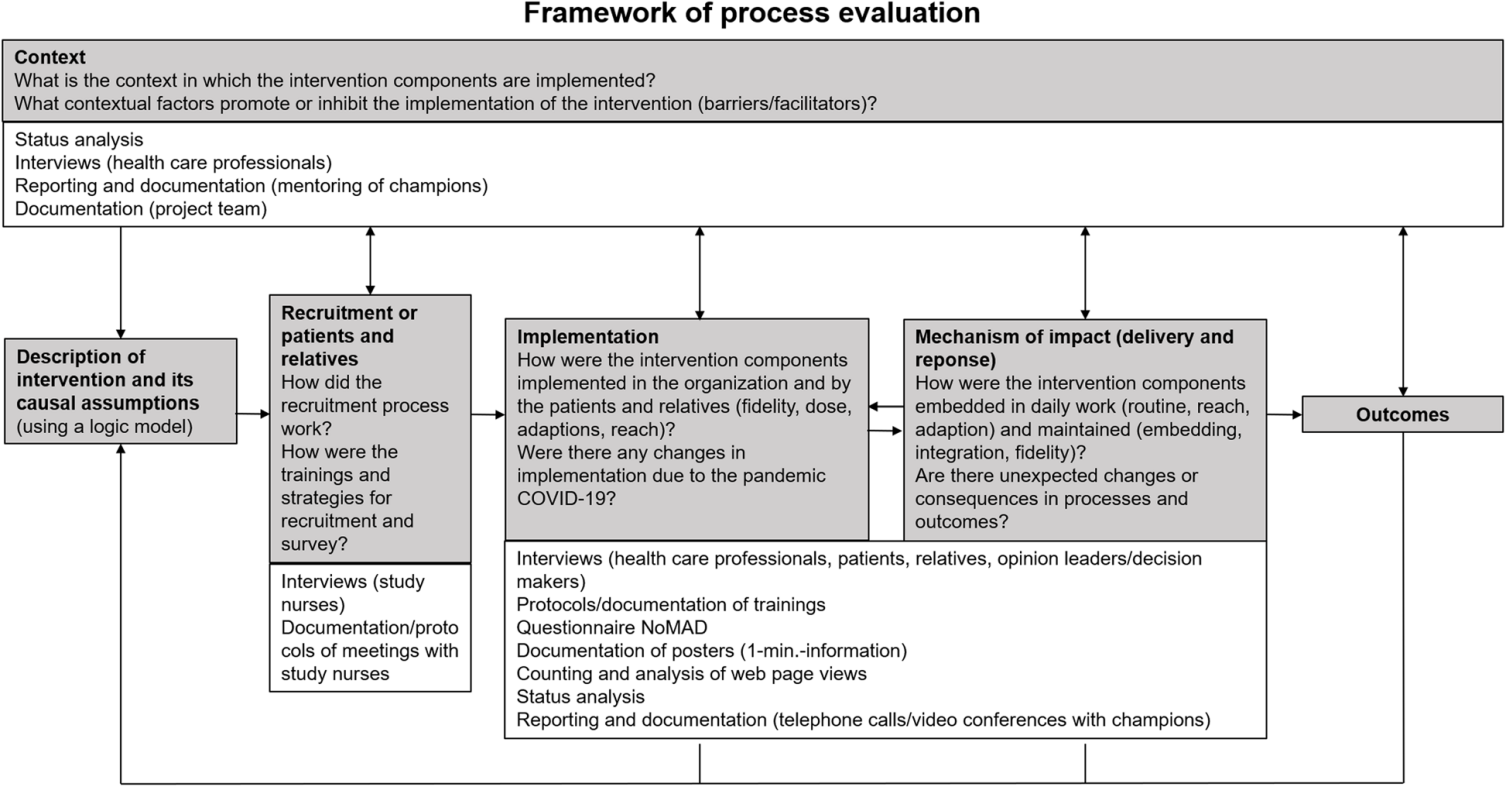
Framework of process evaluation, adapted from Moore et al. [57]. Legend: Grey boxes: Focus of process evaluation and key questions; white boxes: Type of data collection

Based on a developed logic model, research questions for the process evaluation were selected and a mixed-methods study was framed. This mixed-methods study with qualitative and quantitative methods covers all stages of the TRADE study and addresses all stake holders (different health care professionals, patients, caregivers and study nurses). In addition, process indicators, such as use of information material, website access, telephone contacts etc. were used. Figure 5 provides an overview of the process evaluation with the main questions and instruments used. Since both patients and relatives are targeted by the intervention, we focus also on the recruitment of either group to identify possible barriers or facilitators in the process [57].

The Normalization MeAsure Development questionnaire (NoMAD) will be used as a quantitative instrument investigating the integration, embedding and implementation of intervention components in routine practice. The questionnaire consists of 23 items based on NPT [60, 61].

In addition, SARS CoV2 pandemic and its impact on intervention components and implementation will be examined in the evaluation by interviews and status analysis.

An overview of process evaluation shows Fig. 5:

### Ethics

An approval of the regional ethics committee of each center has been obtained prior to the start of recruitment. The study has been registered at the German clinical trials registry (DRKS – Deutsches Register für klinische Studien). All relevant changes will be communicated to the regional ethics committees and the clinical trials registry.

### Dissemination policy

All publications related to the three phases of the TRADE Project will be submitted to international journals after approval from the publication committee of the study. No professional writers are involved in the publication process. Authorship eligibility criteria are set up by the publication committee and comply with International Committee of Medical Journal Editors (ICMJE) statements [62].

A main result will be an established complex intervention with structured training schedules and information materials for health care professionals. The complex intervention is planned to be standardized and further evaluated to be adaptable for other acute care hospitals.

## Discussion

The TRADE study aims to determine the risk of delirium and cognitive decline during and after hospital discharge and transfer in older adults. To our knowledge TRADE will be the first study to prospectively address delirium incidence in the context of transfer, transportation and discharge. TRADE will additionally develop an easily scalable intervention targeting the participation of the primary caregivers to reduce delirium incidence in this context with an indirect train the trainer approach and test its feasibility and acceptance in a pilot clinical trial including a thorough process evaluation.

Non-pharmacologic interventions for delirium are staff- and time-intensive [10, 16, 63]. Still, HELP has proven to be clinical- and cost-efficient in many health care systems across the world, although inclusion of volunteering personnel was an important factor for cost effectiveness [64]. Nevertheless, widespread implementation in regular care is still lacking. Probable reasons are underestimation of long term delirium impact, the considerable staff investment, lack of knowledge concerning effective preventive strategies, difficulties in estimating the health economic cost of delirium [65, 66]. Involvement of caregivers could reduce the need of volunteer-based prevention strategies and thereby health care costs.

A recent review highlights the lack of knowledge of caregivers on delirium and recommends them to play a bigger role in further programs on delirium management [67]. Family HELP (FAM-HELP) is an adaption of the original HELP program involving family members in HELP intervention protocols, that has been investigated in a very small sample (*n* = 15) [68]. In this study caregivers could administer several protocols with a good completion rate of 55 to 84% of HELP protocols (Orientation visit, therapeutic activities, early mobilization, vision protocol, hearing protocol, oral volume repletion/feeding assistance and sleep enhancement) compared to administration by trained volunteers. The Nurse/Family Caregiver Partnership for Delirium Prevention (NFCPM) feasibility study (*n* = 28) investigated an educational program that concurrently taught family caregivers and nurses about delirium and partnering in prevention [69]. The intervention group significantly improved knowledge of delirium and attitudes towards partnership among caregivers as well as nurses. Key to satisfaction in the nurse-caregiver relationship from the caregiver perspective were participation in decision making, communication and respect.

As these studies show, integration of caregivers in delirium prevention strategies merits further investigation. In this process special attention has to be given to communication techniques and interpersonal relationship that are crucial for successful knowledge transfer. In TRADE we aim to set up a multimodal educational program to empower discharge managers and other health care professionals for the successful transfer of knowledge as well as patient’s caregivers.

If the study confirms the hypothesis, our results would add evidence to the question whether change of location during hospital stay and/or related to discharge is another relevant risk factor for the onset of delirium. In addition, the results of the interventional trial will show if the implementation of a multi-component intervention strategy driven by primary caregivers i) is feasible and accepted and ii) can have an effect in the prevention of delirium and cognitive decline after discharge. Following confirmatory studies will have to investigate the effect of such an intervention on delirium incidence reduction, scalability to other acute care hospitals and cost-effectiveness.

## Supplementary Information

**Additional file 1 **: **Supplementary Figure 1.** Algorithm for inclusion or exclusion of participants with evident or suspected cognitive deficits.

## Data Availability

The datasets generated during and/or analyzed during the current study are available from the corresponding author on reasonable request.

## Abbreviations

3D-CAM: 3-min diagnostic interview for Confusion assessment method
ADL: Activities of daily living
AOK: Allgemeine Ortskrankenkasse (public health insurance company)
BCS: The bedside confusion scale
CAM-S: CAM-based scoring system for assessing delirium severity
CCI: Charlson’ s comorbidity index
CI: Confidence interval
CSHA Clinical Frailty Scale: Clinical Frailty Scale of the Canadian Study of Health and Aging
DRKS: Deutsches Register für klinische Studien (German clinical trial registry)
eCRF: Electronic Case Report Form
EDC: Electronic data capture
FAM-CAM: Family Confusion assessment method
FAM-HELP: Family HELP
G-BA: Gemeinsamer Bundesausschuss (Federal Joint Committee)
HELP: Hospital Elder Life Program
IADL: Instrumental activities of daily living
I-CAM: I-Confusion Assessment Method
ICMJE: International Committee of Medical Journal Editors
ICU: Intensive care unit
IQCODE: Informant Questionnaire on Cognitive Decline in the Elderly
MNA-SF: Mini Nutritional Assessment Short Form
MoCA: Montreal Cognitive Assessment
MRC: Medical research council
NPT: Normalization process theory
NRS Pain: Numerical Rating Scale of Pain
NuDESC: Nursing Delirium Screening Scale
PHQ-4: Patient Health Questionnaire
PSQI: Pittsburgh Sleep Quality Index
SARS-CoV2: Severe acute respiratory syndrome coronavirus 2
SAS: Statistical Analysis Software
SSL: Secure Sockets Layer
SQiD: Single question in delirium assessment
TRADE: TRAnsport and DElirium in older people.

## References

[1] Marcantonio ER (2017). Delirium in hospitalized older adults. N Engl J Med.

[2] Witlox J, Eurelings LSM, Jonghe JFM, Kalisvaart KJ, Eikelenboom P, van Gool WA (2010). Delirium in elderly patients and the risk of postdischarge mortality, institutionalization, and dementia: a meta-analysis. JAMA..

[3] Inouye SK (2006). Delirium in Older Persons. N Engl J Med..

[4] Evensen S, Saltvedt I, Lydersen S, Wyller TB, Taraldsen K, Sletvold O. Environmental factors and risk of delirium in geriatric patients: an observational study. BMC Geriatr. 2018;18. 10.1186/s12877-018-0977-y. 10.1186/s12877-018-0977-y PMC6238358 30442109

[5] O’Sullivan R, Inouye SK, Meagher D (2014). Delirium and depression: inter-relationship and overlap in elderly people. Lancet Psychiatry.

[6] Schmitt EM, Gallagher J, Albuquerque A, Tabloski P, Lee HJ, Gleason L (2019). Perspectives on the delirium experience and its burden: common themes among older patients, their family caregivers, and nurses. Gerontologist..

[7] Sánchez A, Thomas C, Deeken F, Wagner S, Klöppel S, Kentischer F (2019). Patient safety, cost-effectiveness, and quality of life: reduction of delirium risk and postoperative cognitive dysfunction after elective procedures in older adults-study protocol for a stepped-wedge cluster randomized trial (PAWEL Study). Trials..

[8] Sharon K, Inouye Rudi GJ, Westendorp Jane S, Saczynski. Delirium in elderly people. Lancet. 2014;383(9920):911-22. 10.1016/S0140-6736(13)60688-1. 10.1016/S0140-6736(13)60688-1 PMC4120864 23992774

[9] Reston JT, Schoelles KM (2013). In-facility delirium prevention programs as a patient safety strategy: a systematic review. Ann Intern Med.

[10] Weinrebe W, Johannsdottir E, Karaman M, Füsgen I (2016). What does delirium cost? An economic evaluation of hyperactive delirium. Z Gerontol Geriatr.

[11] Goldberg A, Straus SE, Hamid JS, Wong CL (2015). Room transfers and the risk of delirium incidence amongst hospitalized elderly medical patients: a case–control study. BMC Geriatr.

[12] Tsutsui S, Kitamura M, Higashi H, Matsuura H, Hirashima S (1996). Development of postoperative delirium in relation to a room change in the general surgical unit. Surg Today.

[13] Meagher DJ, O’Hanlon D, O’Mahony E, Casey PR (1996). The use of environmental strategies and psychotropic medication in the management of delirium. Br J Psychiatry.

[14] McCusker J, Cole MG, Voyer P, Vu M, Ciampi A, Monette J (2013). Environmental factors predict the severity of delirium symptoms in long-term care residents with and without delirium. J Am Geriatr Soc.

[15] Inouye SK, Bogardus ST, Baker DI, Leo-Summers L, Cooney LM (2000). The Hospital Elder Life Program: a model of care to prevent cognitive and functional decline in older hospitalized patients. Hospital Elder Life Program. J Am Geriatr Soc.

[16] Hshieh TT, Yue J, Oh E, Puelle M, Dowal S, Travison T (2015). Effectiveness of multicomponent nonpharmacological delirium interventions: a meta-analysis. JAMA Intern Med.

[17] Craig P, Dieppe P, Macintyre S, Michie S, Nazareth I, Petticrew M (2008). Developing and evaluating complex interventions: the new Medical Research Council guidance. BMJ..

[18] Skivington K, Matthews L, Simpson SA, Craig P, Baird J, Blazeby JM, Boyd KA, Craig N, French DP, McIntosh E, Petticrew M, Rycroft-Malone J, White M, Moore L. A new framework for developing and evaluating complex interventions: update of Medical Research Council guidance. BMJ. 2021:n2061. 10.1136/bmj.n2061. 10.1136/bmj.n2061 PMC8482308 34593508

[19] Wilkes L (2015). Using the Delphi technique in nursing research. Nurs Stand.

[20] Bleijenberg N, de Man-van Ginkel JM, Trappenburg JCA, Ettema RGA, Sino CG, Heim N (2018). Increasing value and reducing waste by optimizing the development of complex interventions: enriching the development phase of the Medical Research Council (MRC) framework. Int J Nurs Stud.

[21] Schmidt B, Krüger L (2016). Lernen in nur einer Minute. Intensiv.

[22] Krüger L, Mannebach T, Wefer F, Bolte C (2021). One Minute Wonder – Fortbildung während der Arbeitszeit. HBScience..

[23] Hendlmeier I, Bickel H, Hessler JB, Weber J, Junge MN, Leonhardt S (2018). Demenzsensible Versorgungsangebote im Allgemeinkrankenhaus: Repräsentative Ergebnisse aus der General Hospital Study (GHoSt) Dementia friendly care services in general hospitals: Representative results of the general hospital study (GHoSt). Z Gerontol Geriatr.

[24] Fong TG, Davis D, Growdon ME, Albuquerque A, Inouye SK (2015). The interface between delirium and dementia in elderly adults. Lancet Neurol.

[25] Goldberg TE, Chen C, Wang Y, Jung E, Swanson A, Ing C (2020). Association of delirium with long-term cognitive decline: a meta-analysis. JAMA Neurol.

[26] Hestermann U, Backenstrass M, Gekle I, Hack M, Mundt C, Oster P (2009). Validation of a German version of the confusion assessment method for delirium detection in a sample of acute geriatric patients with a high prevalence of dementia. Psychopathology..

[27] Inouye SK, van Dyck CH, Alessi CA, Balkin S, Siegal AP, Horwitz RI (1990). Clarifying confusion: the confusion assessment method. A new method for detection of delirium. Ann Intern Med.

[28] Thomas C, Kreisel SH, Oster P, Driessen M, Arolt V, Inouye SK (2012). Diagnosing delirium in older hospitalized adults with dementia: adapting the confusion assessment method to international classification of diseases, tenth revision, diagnostic criteria. J Am Geriatr Soc.

[29] Inouye SK, Kosar CM, Tommet D, Schmitt EM, Puelle MR, Saczynski JS (2014). The CAM-S: development and validation of a new scoring system for delirium severity in 2 cohorts. Ann Intern Med.

[30] Marcantonio ER, Ngo LH, O’Connor M, Jones RN, Crane PK, Metzger ED (2014). 3D-CAM: derivation and validation of a 3-minute diagnostic interview for CAM-defined delirium: a cross-sectional diagnostic test study. Ann Intern Med.

[31] Steis MR, Evans L, Hirschman KB, Hanlon A, Fick DM, Flanagan N (2012). Screening for delirium via family caregivers: convergent validity of the family confusion assessment method (FAM-CAM) and interviewer-rated CAM. J Am Geriatr Soc.

[32] Brich J, Baten V, Wußmann J, Heupel-Reuter M, Perlov E, Klöppel S (2019). Detecting delirium in elderly medical emergency patients: validation and subsequent modification of the German nursing delirium screening scale. Intern Emerg Med.

[33] Stillman MJ, Rybicki LA (2000). The bedside confusion scale: development of a portable bedside test for confusion and its application to the palliative medicine population. J Palliat Med.

[34] Sands MB, Dantoc BP, Hartshorn A, Ryan CJ, Lujic S (2010). Single question in delirium (SQiD): testing its efficacy against psychiatrist interview, the confusion assessment method and the memorial delirium assessment scale. Palliat Med.

[35] Nasreddine ZS, Phillips NA, Bédirian V, Charbonneau S, Whitehead V, Collin I (2005). The Montreal cognitive assessment, MoCA: a brief screening tool for mild cognitive impairment. J Am Geriatr Soc..

[36] Mahoney FI, Barthel D (1965). Functional evaluation: the Barthel index. Md State Med J..

[37] Lübke N, Meinck M, Renteln-Kruse W. Der Barthel-Index in der Geriatrie. Eine Kontextanalyse zum Hamburger Einstufungsmanual. Z Gletscherk Glazialgeol. 2004;37:316–26. 10.1007/s00391-004-0233-2 15338161

[38] Lawton MP, Brody EM (1969). Assessment of older people: self-maintaining and instrumental activities of daily living. Gerontologist.

[39] Collen FM, Wade DT, Robb GF, Bradshaw CM (1991). The Rivermead mobility index: a further development of the Rivermead motor assessment. Int Disabil Stud.

[40] Pasina L, Colzani L, Cortesi L, Tettamanti M, Zambon A, Nobili A (2019). Relation between delirium and anticholinergic drug burden in a cohort of hospitalized older patients: an observational study. Drugs Aging.

[41] Boustani M, Campbell N, Munger S, Maidment I, Fox C (2008). Impact of anticholinergics on the aging brain: a review and practical application. Aging Health.

[42] Ricci F, Cedrone C, Cerulli L (1998). Standardized measurement of visual acuity. Ophthalmic Epidemiol.

[43] Pirozzo S, Papinczak T, Glasziou P (2003). Whispered voice test for screening for hearing impairment in adults and children: systematic review. BMJ..

[44] Phillips P (1986). Grip strength, mental performance and nutritional status as indicators of mortality risk among female geriatric patients. Age Ageing.

[45] Geerlings MI, Jonker C, Bouter LM, Adèr HJ, Schmand B (1999). Association between memory complaints and incident Alzheimer’s disease in elderly people with normal baseline cognition. Am J Psychiatry..

[46] Charlson ME, Pompei P, Ales KL, MacKenzie CR (1987). A new method of classifying prognostic comorbidity in longitudinal studies: development and validation. J Chronic Dis..

[47] Kroenke K, Spitzer RL, Williams JB, Löwe B (2009). An ultra-brief screening scale for anxiety and depression: the PHQ-4. Psychosomatics..

[48] Buysse DJ, Reynolds CF, Monk TH, Berman SR, Kupfer DJ (1989). The Pittsburgh sleep quality index: a new instrument for psychiatric practice and research. Psychiatry Res..

[49] Rockwood K, Song X, MacKnight C, Bergman H, Hogan DB, McDowell I (2005). A global clinical measure of fitness and frailty in elderly people. CMAJ..

[50] Hawker GA, Mian S, Kendzerska T, French M. Measures of adult pain: visual analog scale for pain (VAS pain), numeric rating scale for pain (NRS pain), McGill pain questionnaire (MPQ), short-form McGill pain questionnaire (SF-MPQ), chronic pain grade scale (CPGS), short Form-36 bodily pain scale (SF-36 BPS), and measure of intermittent and constant osteoarthritis pain (ICOAP). Arthritis Care Res. 2011;63 Suppl 11:S240–52. 10.1002/acr.20543 22588748

[51] Jorm AF (1994). A short form of the informant questionnaire on cognitive decline in the elderly (IQCODE): development and cross-validation. Psychol Med.

[52] Wellek S, Donner-Banzhoff N, König J, Mildenberger P, Blettner M (2019). Planning and analysis of trials using a stepped wedge design. Dtsch Arztebl Int.

[53] Thompson JA, Davey C, Fielding K, Hargreaves JR, Hayes RJ (2018). Robust analysis of stepped wedge trials using cluster-level summaries within periods. Stat Med.

[54] Goldfeld K (2020). Analysing an open cohort stepped-wedge clustered trial with repeated individual binary outcomes.

[55] O’Kelly M, Ratitch B (2014). Clinical trials with missing data: a guide for practitioners.

[56] Moore GF, Audrey S, Barker M, Bond L, Bonell C, Hardeman W (2015). Process evaluation of complex interventions: Medical Research Council guidance. BMJ..

[57] May C, Finch T, Mair F, Ballini L, Dowrick C, Eccles M (2007). Understanding the implementation of complex interventions in health care: the normalization process model. BMC Health Serv Res.

[58] May C, Finch T (2009). Implementing, embedding, and integrating practices: an outline of normalization process theory. Sociology..

[59] Grant A, Treweek S, Dreischulte T, Foy R, Guthrie B (2013). Process evaluations for cluster-randomised trials of complex interventions: a proposed framework for design and reporting. Trials..

[60] Rapley T, Girling M, Mair FS, Murray E, Treweek S, McColl E (2018). Improving the normalization of complex interventions: part 1 - development of the NoMAD instrument for assessing implementation work based on normalization process theory (NPT). BMC Med Res Methodol.

[61] Finch TL, Girling M, May CR, Mair FS, Murray E, Treweek S (2018). Improving the normalization of complex interventions: part 2 - validation of the NoMAD instrument for assessing implementation work based on normalization process theory (NPT). BMC Med Res Methodol.

[62] International Committee of Medical Journal Editors. Defining the role of authors and contributors. http://www.icmje.org/recommendations/browse/roles-and-responsibilities/defining-the-role-of-authors-and-contributors.html. Accessed 1 May 2018. http://www.icmje.org/recommendations/browse/roles-and-responsibilities/defining-the-role-of-authors-and-contributors.html.

[63] Leslie DL (2008). One-year health care costs associated with delirium in the elderly population. Arch Intern Med.

[64] Hshieh TT, Yang T, Gartaganis SL, Yue J, Inouye SK (2018). Hospital elder life program: systematic review and meta-analysis of effectiveness. Am J Geriatr Psychiatry.

[65] Caplan GA, Teodorczuk A, Streatfeild J, Agar MR (2020). The financial and social costs of delirium. Eur Geriatr Med.

[66] Singler K, Thomas C (2017). HELP - Hospital Elder Life Program - multimodal delirium prevention in elderly patients. Internist (Berl).

[67] Shrestha P, Fick DM (2020). Family caregiver’s experience of caring for an older adult with delirium: a systematic review. Int J Older People Nursing.

[68] Rosenbloom-Brunton DA, Henneman EA, Inouye SK (2010). Feasibility of family participation in a delirium prevention program for hospitalized older adults. J Gerontol Nurs.

[69] Rosenbloom DA, Fick DM (2014). Nurse/family caregiver intervention for delirium increases delirium knowledge and improves attitudes toward partnership. Geriatr Nurs.

