# Supplementary figures and images for: A complex intervention to promote prevention of delirium in older adults by targeting caregiver’s participation during and after hospital discharge – study protocol of the TRAnsport and DElirium in older people (TRADE) project
Source: BMC Geriatr. 2021 Nov 16;21:646. doi: 10.1186/s12877-021-02585-0 (PMC8594294; doi:10.1186/s12877-021-02585-0)

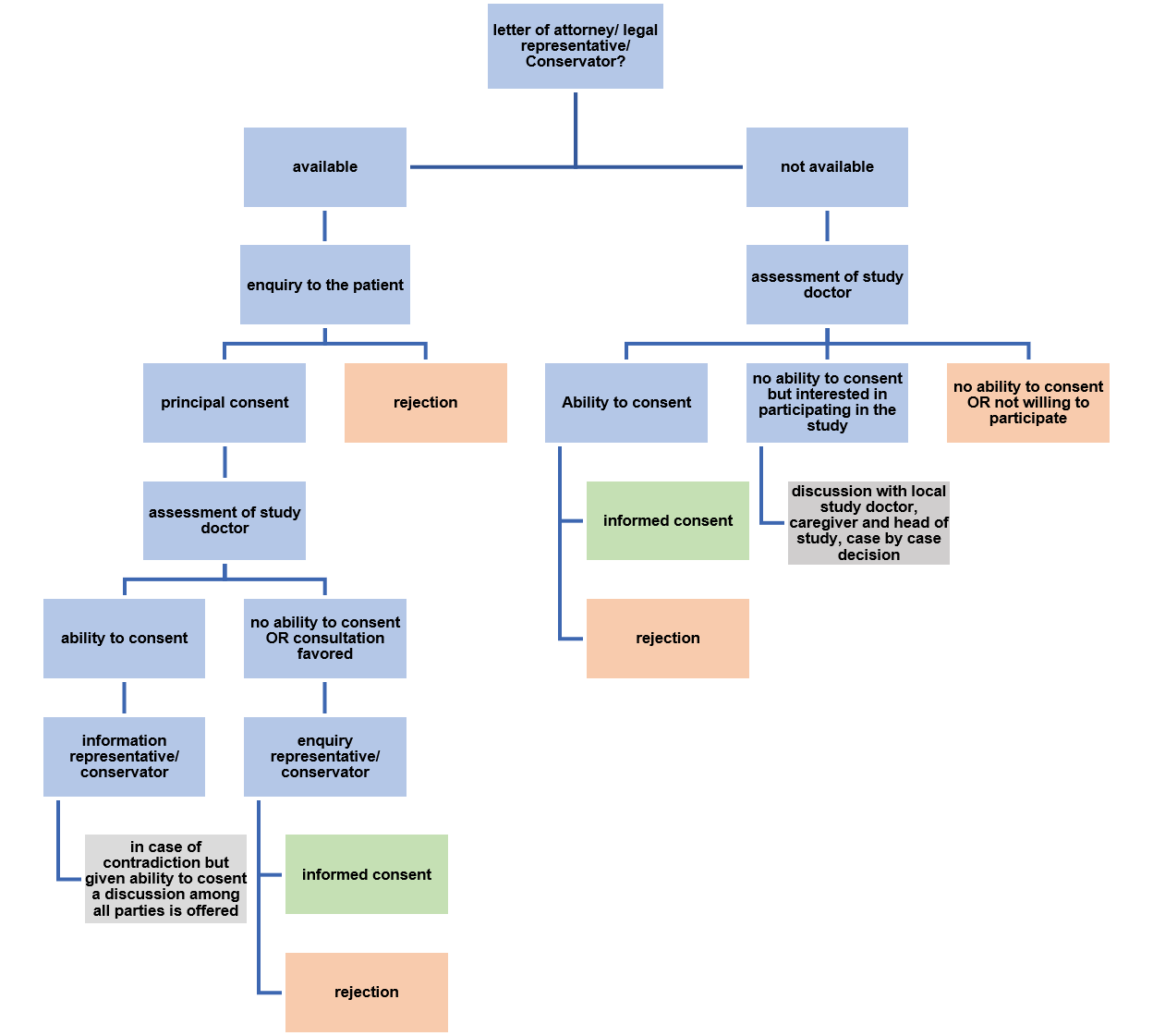

Supplement: Supplementary file 1 — Additional file 1 : Supplementary Figure 1. Algorithm for inclusion or exclusion of participants with evident or suspected cognitive deficits. [file 12877_2021_2585_MOESM1_ESM.png]
